# RAS-MAPK pathway epigenetic activation in cancer: miRNAs in action

**DOI:** 10.18632/oncotarget.6476

**Published:** 2015-12-05

**Authors:** Julien Masliah-Planchon, Simon Garinet, Eric Pasmant

**Affiliations:** ^1^ Unité de Génétique Somatique, Département de Génétique Oncologique, Institut Curie, Paris, France; ^2^ INSERM_U830, Institut Curie, Paris, France; ^3^ Service de Biochimie et Génétique Moléculaire, Hôpital Cochin, Assistance Publique-Hôpitaux de Paris, Paris, France; ^4^ EA7331, Université Paris Descartes, Sorbonne Paris Cité, Faculté des Sciences Pharmaceutiques et Biologiques, Paris, France

**Keywords:** RAS MAPK pathway, microRNAs, cancer, epigenetics

## Abstract

The highly conserved RAS-mitogen activated protein kinase (MAPK) signaling pathway is involved in a wide range of cellular processes including differentiation, proliferation, and survival. Somatic mutations in genes encoding RAS-MAPK components frequently occur in many tumors, making the RAS-MAPK a critical pathway in human cancer. Since the pioneering study reporting that let-7 miRNA acted as tumor suppressor by repressing the *RAS* oncogene, growing evidence has suggested the importance of miRNAs targeting the RAS-MAPK in oncogenesis. MiRNAs alterations in human cancers may act as a rheostat of the oncogenic RAS signal that is often amplified as cancers progress. However, specific mechanisms leading to miRNAs deregulation and their functional consequences in cancer are far from being fully elucidated. In this review, we provide an experimental-validated map of RAS-MAPK oncomiRs and tumor suppressor miRNAs from transmembrane receptor to downstream ERK proteins. MiRNAs could be further considered as potential genetic biomarkers for diagnosis, prognosis, or therapeutic purpose.

## RAS-MAPK PATHWAY IS MUTATED IN HUMAN CANCER

Function of the RAS-Mitogen activated protein kinase (MAPK) signaling pathway (also known as the RAS-RAF-MEK-ERK pathway) is to integrate extracellular signals and coordinate a suitable response by a subsequent control of cellular growth, survival, and differentiation. Aberrant activation of this pathway is a major and highly prevalent oncogenic event in many human cancers. Oncogenic RAS mutations occur in approximately 30% of all tumor types; however, mutations in upstream regulators and downstream effectors are also prevalent [1]. As an example, *KRAS* (*V-Ki-ras2 Kirsten rat sarcoma viral oncogene homolog*) somatic mutations were identified in more than 90% of pancreatic ductal adenocarcinoma (PDAC) [2]; *BRAF* and *NRAS* mutations occur with mutual exclusion in melanoma and account altogether for 83% of the cases [3]; RAS-MAPK signaling pathway is altered in 67% of the T-cell precursor acute lymphoblastic leukemia including mutations in *NRAS*, *KRAS*, *BRAF*, *NF1*, and *PTPN11* [4]; 55% of colon and rectal cancer have alterations in *KRAS*, *NRAS,* or *BRAF* [5]; *KRAS*, *EGFR*, *NF1*, and *BRAF* mutations occur in 27%, 17%, 11%, and 3% in lung adenocarcinomas, respectively [6, 7].

## RAS-MAPK PATHWAY IS EPIGENETICALLY ALTERED IN HUMAN CANCER: MIRNAS IN ACTION

It is likely that mechanisms other than mutations contribute to RAS-MAPK pathway activation in cancer. Recently, epigenetic alterations were described to potentiate this activation in human tumors [8]. Epigenetic processes contribute to the regulation of gene expression and have a critical role in cell fate specification. The past decade has highlighted the increasing role of epigenetic in oncogenesis. Epigenetic modifications refer to heritable genomic changes in the absence of alterations in the DNA sequence through covalent histone-tail modifications, DNA methylation, chromatin remodeling, and regulation of non-coding RNA expression. Non-coding RNAs, including microRNAs (miRNAs), promote the establishment and maintenance of an epigenetic state and contribute to gene expression homeostasis. In addition, epigenetic factors can be responsible for the dysregulation of the miRNome (defined as the full spectrum of miRNAs expression) observed in cancer. Conversely, some miRNAs directly target the epigenetic machinery and subsequently affect the expression of tumor suppressor genes or oncogenes [9]. MiRNAs are small (~ 22 nucleotides long) highly conserved noncoding RNAs that epigenetically target mRNAs and subsequently repress protein expression. MiRNAs are involved in a very wide variety of cellular processes. Very few miRNAs have been described to be causative for heritable diseases [10–12]. Conversely, numerous miRNAs have been described to be differentially expressed in cancer: either up-regulated and acting as oncogenes (commonly termed oncomiRs) or down-regulated and acting as tumor suppressor miRNAs [13, 14].

**Figure 1:**
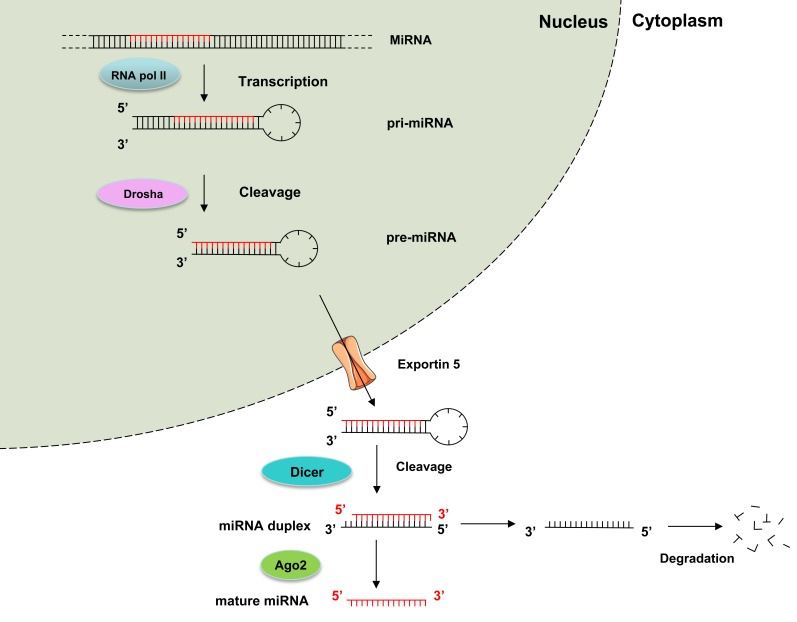
The canonical miRNA processing pathway includes the production of the primary miRNA transcript (pri-miRNA) by RNA polymerase II and cleavage of the pri-miRNA by the microprocessor complex Drosha in the nucleus. The resulting precursor hairpin, the pre-miRNA, is exported from the nucleus by Exportin-5. In the cytoplasm, the RNase Dicer cleaves the pre-miRNA hairpin to its mature length. The functional strand of the mature miRNA is loaded together with Argonaute (Ago2) proteins into the RNA-induced silencing complex (RISC), where it guides RISC to silence target mRNAs through mRNA cleavage, translational repression, whereas the passenger strand is degraded [15].

MiRNAs biogenesis implies a multistep process that starts in the nucleus with the transcription of several kilobases (kb) precursors of miRNA (pri-miRNAs) by RNA polymerase II (Figure 1) [15]. Pri-miRNAs are then cleaved in the nucleus by Drosha ribonuclease into hairpin-structured 60-100 nucleotides long pre-miRNAs. Pre-miRNA hairpins recognition by exportin 5 enables nuclear export and final processing in the cytoplasm by Dicer ribonuclease to generate a ~22-nucleotides double-stranded miRNA. One strand of the mature miRNA duplex selectively associates with argonaute (Ago) proteins to form the RNA-induced silencing complex (RISC). RISC binds predominantly to the 3′ untranslated region (UTR) of messenger RNAs (mRNAs) through the miRNA seed sequence and finally represses gene expression either by reducing translation or by stimulating mRNAs decay.

Each miRNA putatively regulates expression of thousands of different protein-coding genes and one specific mRNA can be targeted by hundreds of miRNAs. A large variety of bioinformatics tools have been developed to reliably predict mRNA targets of a specific miRNA. However, the results of these computational tools are frequently quite different from one to another, if not contradictory. Effective prediction of miRNA targets still remains challenging and must be experimentally validated by molecular assays (such as luciferase assay).

Since their initial discovery in the 90's, mounting evidence has demonstrated that miRNAs have an important role in tumor formation, progression, and resistance to therapy [14]. In 2009, germline inactivating *DICER1* mutations have been associated with familial pleuropulmonary blastoma, a rare malignant lung tumor mainly affecting children [16]. The highly conserved Dicer endoribonuclease is essential for the production of miRNAs (Figure 1) [15, 17]. In addition to pleuropulmonary blastoma, germline *DICER1* mutations also predispose to a large variety of different rare tumors. Somatic mutation of *DICER1* and *DROSHA*, another ribonuclease involved in miRNA biogenesis, have also been described in tumors such as ovarian cancer [18] and Wilms tumor [19]. MiRNA processing deficiency promoted by somatic alterations of *DICER1* or *DROSHA* genes probably support tumor cell proliferation. Although it is likely that alterations in *DICER1* and *DROSHA* genes globally disturb miRNA processing, the precise effect on miRNome is not well known. However, we can assume that large miRNA families such as let-7 family should be affected by *DICER1* and *DROSHA* somatic mutations. In addition, it has been demonstrated that let-7 directly affects *DICER1* expression by targeting its 3′UTR [20]. This suggests the possible existence of a regulatory loop, in which let-7 may play a role for regulating the level of dicer.

**Figure 2:**
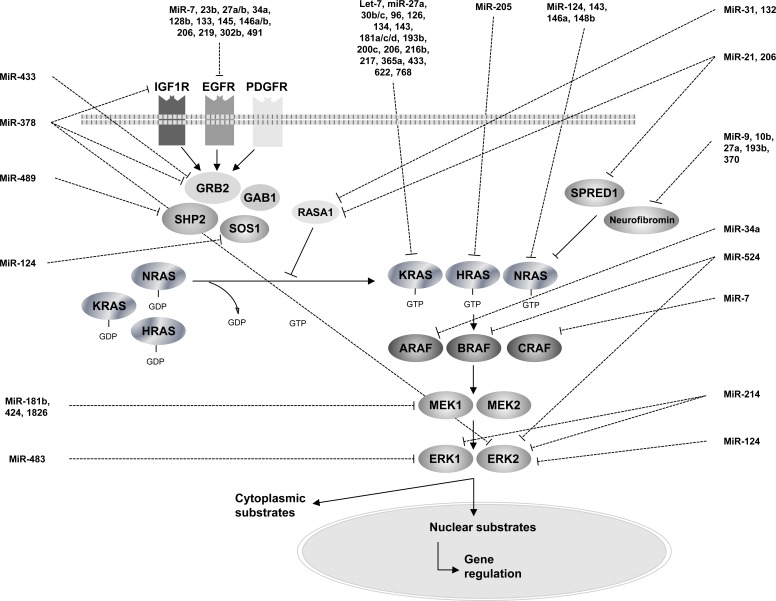
An overview of RAS-MAPK pathway regulation by microRNAs

Various miRNAs have been demonstrated to target members of the RAS-MAPK pathway. Hence, deregulation of such miRNAs in cancer cells most likely contributes to tumorigenesis by leading to an aberrant activation of the RAS-MAPK pathway. In the present manuscript, we review the miRNAs that have been experimentally proven to epigenetically target members of the RAS-MAPK signaling pathway in human cancer: we provide an experimental-validated map of RAS-MAPK control by oncomiRs and suppressor miRNAs from transmembrane receptors to ERK proteins (Figure 2, Table 1).

**Table 1:** Control of RAS-MAPK pathway by microRNAs in cancer

RAS-MAPK target	miRNA	Expression variation and Tumor context
*EGFR*	miR-128b	↘ in non-small-cell lung cancer
*EGFR*	miR-145	↘ in lung adenocarcinoma
*EGFR*	miR-206	↘ in lung squamous cell carcinoma
*EGFR*	miR-219-5p	↘ in glioblastoma
*EGFR*	miR-7	↘ in glioblastoma
*EGFR*	miR-146b-5p	↘ in glioblastoma
*EGFR*	miR-34a	↘ in glioblastoma
*EGFR*	miR-27a-5p	↘ in head and neck squamous cell carcinoma
*EGFR*	miR-302b	↘ in hepatocellular carcinoma
EGFR	miR-491-5p	↘ in ovarian carcinoma
*EGFR*	miR-23b/27b	↘ in bladder carcinoma
*EGFR*	miR-133a/b	↘ in hormone-insensitive prostate cancer
*EGFR*	miR-146a	↘ in hormone-insensitive prostate cancer
*EGFR*	miR-133a	↘ in breast cancer
*GRB2*	miR-433	↘ in gastric carcinoma
*GRB2*	miR-378	↘ in gastric carcinoma↘ in prostate cancer
*PTPN11*	miR-489	↘ in hypopharyngeal squamous cell carcinoma
*SOS*	miR-124	↘ in glioblastoma
*KRAS*	let-7	↘ in many cancer types (reviewed by Boyerinas et al., 2010 [48])
*KRAS*	miR-181a	↘ in oral squamous cell carcinoma
*KRAS*	miR-181c	↘ in gastric carcinoma
*KRAS*	miR-181d	↘ in glioma
*KRAS*	miR-134	↘ in glioblastoma↘ in renal cell carcinoma
*KRAS*	miR-126	↘ in pancreatic ductal adenocarcinoma
*KRAS*	miR-96	↘ in pancreatic cancer
*KRAS*	miR-217	↘ in pancreatic ductal adenocarcinoma
*KRAS*	miR-143	↘ in colorectral cancer
*KRAS*	miR-200c	↘ in breast and lung cancer cell lines
*KRAS*	miR-216b	↘ in nasopharyngeal carcinoma
*KRAS*	miR-622	↘ in a model of transformed human bronchial epithelial cell line
*KRAS*	miR-433	↘ in gastric carcinoma
*KRAS*	miR-768	↘ in brain metastase compared to primary tumor
*KRAS*	miR-27a	↘ in esophageal squamous cell carcinoma
*KRAS*	miR-30b	↘ in colorectral cancer
*KRAS*	miR-30c	↘ in breast cancer
*KRAS*	miR-193b/365a	↘ in a mouse skin tumorigenesis model
*KRAS*	miR-206	↘ in pancreatic ductal adenocarcinoma
*NRAS*	miR-148b	↘ in breast cancer
*NRAS*	miR-124	↘ in glioblastoma
*NRAS*	miR-143	↘ in glioma
*HRAS*	miR-205	↘ in prostate carcinoma
*BRAF*	miR-524-5p	↘ in melanoma
*ARAF*	miR-34a	↘ in many cancer types (reviewed by Li et al., 2014 [102])
*RAF1*	miR-7	↘ in lung, breast, and glioblastoma cell lines
*MEK1*	miR-424	↘ in senile hemangioma
*MEK1*	miR-1826	↘ in bladder cancer and VHL-inactivated renal cancer
*MEK1*	miR-181b	↘ in glioma
*MAP2K4*	miR-181a	↘ in pancreatic cancer
*ERK1*	miR-483-5p	↘ in glioma
*ERK1*	miR-214	↘ in squamous cell carcinoma
*ERK2*	miR-214	↘ in squamous cell carcinoma
*ERK2*	miR-124	↘ in squamous cell carcinoma
*ERK2*	miR-524-5p	↘ in melanoma
*SPRED1*	miR-21	↗ enhanced triple-negative breast cancer
*SPRED1*	miR-206	↗ enhanced triple-negative breast cancer
*RASA1*	miR-21	↗ enhanced triple-negative breast cancer
*RASA1*	miR-206	↗ enhanced triple-negative breast cancer
*RASA1*	miR-132	↗ in endothelium of many tumors
*RASA1*	miR-31	↗ in colorectal cancer
*NF1*	miR-10b	↗ in malignant peripheral nerve sheath tumors
*NF1*	miR-27a	↗ in T-cell acute lymphoblastic leukemia
*NF1*	miR-370	↗ in acute myeloid leukemia
*NF1*	miR-9	↗ in glioma
*NF1*	miR-193	↗ in head and neck squamous cell carcinomas

## MIRNAS DYSREGULATION ALONG THE RAS-MAPK PATHWAY IN HUMAN CANCER

The RAS-MAPK pathway initiates with growth-factor binding to cell-surface receptors. Receptor tyrosine kinases (RTKs), a specific category of transmembrane receptors, can translate a ligand-binding event into a signal and can ultimately cause a wide array of cellular responses, including cell growth, differentiation, division, adhesion, migration, and apoptosis. One subfamily of RTKs contains the erythroblastic leukemia viral oncogene homologue (ERBB) receptors. In several malignancies, including lung adenocarcinomas, ductal carcinomas of the breast, and glioblastoma (GBM), ERBB receptors signalings are deregulated, driving uncontrolled proliferation of tumor cells, conferring the ability to evade programmed cell death, enhancing their ability to migrate, and facilitating metastasis. This ERBB family of RTKs consists of four receptors: ERBB1, ERBB2, ERBB3, and ERBB4 (also known as HER1, HER2, HER3, and HER4, respectively). ERBB1 is also known as the epidermal growth factor receptor (EGFR).

## MIRNAS TARGETING EGFR

Binding of the receptor to a ligand induces EGFR dimerization, tyrosine autophosphorylation, and ultimately leads to cell proliferation. In the era of molecular and personalized therapeutics, the discovery of mutations in *EGFR* in 15-20% of lung adenocarcinomas and the associated response to EGFR-targeting tyrosine kinase inhibitors have provided a successful avenue of treatment in high-stage lung adenocarcinomas.

Several miRNAs were described as experimentally direct regulators of *EGFR*, acting as tumor suppressors in various tumor types. Identifying microRNA regulators of oncogenes could have far-reaching implications, in particular for lung cancer patients including improving patient selection for targeted agents, development of novel therapeutics, or usage as early biomarkers of disease. MiR-128b was shown to be a direct negative regulator of *EGFR* in non-small-cell lung cancer (NSCLC) cell lines [21]. MiR-128b loss of heterozygosity in chromosome 3p is frequent in NSCLC tumor samples and was significantly correlated with clinical response to targeted EGFR inhibition following gefitinib treatment [21]. Loss of miR-128b would be equivalent to losing a tumor suppressor gene because it would allow increased expression of *EGFR*. MiR-145 was also described as a negative regulator of *EGFR* expression at both mRNA and protein levels in lung adenocarcinoma [22]. Interestingly, miR-145 transfection in lung adenocarcinoma cells inhibited proliferation. Expression of the oncogene hepatocyte growth factor receptor (also called MET) and its phosphorylation was associated with resistance to tyrosine kinase inhibitors used in therapy targeting EGFR in patients with lung carcinomas. Overexpression of *MET* and *EGFR* and down-regulation of miR-206 were observed in clinical specimens of lung squamous cell carcinoma (SCC) [23]. Restoration of mature miR-206 inhibited cancer cell proliferation, migration, and invasion in human lung SCC cell lines through down-regulation of both mRNA and protein levels of MET and EGFR. Interestingly, phosphorylation of ERK1/2 and AKT downstream effectors were inhibited by restoration of miR-206 in cancer cells, indicating that tumor-suppressive miR-206 inhibited dual signaling networks activated by MET and EGFR.

Up to 45% of GBM show amplifications and activating mutations in the *EGFR* gene leading to the up-regulation of the RAS-MAPK pathway. The expression of miR-219-5p was shown to be down-regulated in GBM and the overexpression of miR-219-5p in GBM cell lines inhibited the proliferation, anchorage independent growth and migration [24]. A significant negative correlation between miR-219-5p levels and total as well as phosphorylated forms of EGFR was found in GBM patient samples. MiR-219-5p inhibited RAS-MAPK and PI3K pathways in GBM cell lines in concordance with its ability to repress *EGFR* by directly binding to its 3′-UTR. This inhibitory effect could be rescued by the overexpression of wild-type *EGFR*. MiR-7 potently also suppressed *EGFR* expression in GBM, and independently inhibited the AKT pathway [25]. MiR-7 expression was down-regulated in GBM, with a mechanism involving processing defect in generating pre-miR-7 from pri-miR-7. Transfection with miR-7 decreased viability and invasiveness of primary GBM lines. MiR-146b-5p was also described as a negative regulator of *EGFR*, binding to the *EGFR* 3′-UTR. Introduction of miR-146b-5p reduced *in vitro* cell invasion, migration, and phosphorylation of AKT in human GBM cell lines [26]. Interestingly, human miR-146b-5p is located on chromosome 10q24.3, a region that is frequently loss in human GBM [27]. MiR-34a was also described as a negative regulator of *EGFR* [28]. Forced expression of miR-34a in GBM cells decreased their ability to migrate and potently reduced their proliferation by profoundly decreasing their levels of cyclin-dependent kinase and increased expression of p21 and p27 cyclin kinase inhibitor proteins. MiR-34a also targeted the YY1 (Yin Yang-1) transcription factor that can stimulate the expression of *EGFR*. Mean survival time was significantly shortened for patients whose GBM had both *EGFR* amplification and miR-34a deletion with significantly lower miR-34a expression.

EGFR has also been characterized as a critical factor in the development and progression of head and neck squamous cell carcinoma (HNSCC). MiR-27a-3p and its complementary strand miR-27a-5p were significantly down-regulated in multiple HNSCC cell lines [29]. MiR-27a-5p forced increased expression produced a profound cytotoxic effect, with coordinated down-regulation of EGFR, AKT and mTOR. Constitutive and inducible expression of miR-27a-5p in a murine orthotopic xenograft model of oral cavity cancer led to decreased tumor growth and direct intra-tumoral injection of miR-27a-5p inhibited tumor growth *in vivo*.

MiR-302b was frequently down-regulated, whereas *EGFR* was up-regulated in human hepatocellular carcinoma (HCC) [30]. The dual luciferase reporter assay revealed that *EGFR* was a target of miR-302b. Re-expression of miR-302b resulted in the inhibition of proliferation in HCC SMMC-7721 cells. The silencing of *EGFR* by miR-302b or siRNA led to down-regulation of proliferation-related proteins, such as AKT2, CCND1, and CDK2.

MiR-491-5p efficiently induced apoptosis in ovarian carcinoma IGROV1-R10 cell line by inducing pro-apoptotic BIM accumulation in its dephosphorylated form [31]. This effect was due to direct targeting of *EGFR* by miR-491-5p and consequent inhibition of downstream AKT and RAS-MAPK signaling pathways. Induction of apoptosis by miR-491-5p in the IGROV1-R10 cell line was mimicked by a combination of EGFR inhibition together with a BIM-mimetic molecule.

The expression levels of the miR-23b/27b cluster were significantly reduced in advanced bladder cancer clinical specimens [32]. Luciferase reporter assays and western blotting demonstrated that *EGFR* and *MET* transcripts were directly regulated by the miR-23b/27b cluster. The decreased expression of the tumor-suppressive miR-23b/27b cluster enhanced cancer cell proliferation, migration and invasion in bladder cancer through direct regulation of *EGFR* and *MET*. MiR-133 has long been recognized as a muscle-specific miRNA which may regulate myoblast differentiation and participate in many myogenic diseases. Recently, miR-133a and miR-133b were shown to be weakly expressed in two hormone-insensitive prostate cancer cell lines, PC3 and DU145 [33]. Ectopic expression of miR-133 inhibited cell proliferation, migration and invasion in these cells by targeting *EGFR*. Moreover, expression of miR-133a was significantly down-regulated in breast cancer cell lines and tissues [34]. Overexpression of miR-133a in tumor cells arrested the cell cycle by drastically decreasing the G2/S phase and retarded the newly synthesized DNA. A dual luciferase assay showed that miR-133a bound to the wild-type 3′-UTR of *EGFR* but not a mutated 3′-UTR, thereby down-regulating the protein expression level. MiR-146a expression was significantly decreased in castration-resistant prostate cancer tissues compared to androgen-dependent prostate cancer tissues [35]. Ectopic overexpression of miR-146a in androgen-independent cell lines inhibited cell growth, colony formation, and migration *in vitro*, and reduced tumorigenicity and angiogenesis *in vivo*. MiR-146a repressed the expression of *EGFR* through binding to its 3′-UTR region.

Conversely, EGFR could modulate miRNAs formation and maturation. For example, EGFR could suppress the maturation of specific tumor-suppressor miRNAs in response to hypoxic stress through phosphorylation of argonaute 2 (AGO2) [36]. The association between EGFR and AGO2 was shown to be enhanced by hypoxia, leading to elevated AGO2 phosphorylation, which in turn reduced the binding of Dicer to AGO2 and inhibited miRNA processing from precursor miRNAs to mature miRNAs. AGO2 phosphorylation mediated EGFR-enhanced cell survival and invasiveness under hypoxia, and was correlated with poorer overall survival in breast cancer patients. In a recent study, nuclear EGFR was suggested to act as a transcriptional repressor of miR-1 which targets *TWIST*. In prostate cancer, this oncogenic activation of TWIST1 led to an accelerated bone metastasis [37].

## MIRNAS TARGETING *GRB2*

After growth-factor binding to cell-surface receptors, such as EGFR, subsequent intracellular ligation of various adaptors including growth-factor-receptor bound protein 2 (GRB2) contributes to the induction of the RAS-MAPK pathway. MiR-433 was found to be down-regulated in gastric carcinoma compared with normal stomach samples and to target *GRB2* transcript [38]. MiR-378 has also been demonstrated to target *GRB2*. Demonstration has come originally from the observation that miR-378 is strongly overexpressed in cardiomyocytes. MiR-378 overexpression could repress cardiomyocytes proliferation and subsequent cardiac hypertrophy by suppressing the RAS-MAPK signaling pathway [39]. In addition to *GRB2*, miR-378 directly targeted three additional components of the RAS-MAPK pathway: Mitogen-activated protein kinase 1 (*MAPK1*, alias *ERK2*), insulin-like growth factor receptor 1 (*IGF1R*), and kinase suppressor of RAS 1 (*KSR1*) [40]. MiR-378 was found down-regulated in various types of cancers such as prostate [41] and gastric cancers [42]. MiR-433 and miR-378 down-regulation in cancer could hence represent an oncogenic event leading to an aberrant activation of the RAS-MAPK pathway by enhancing GRB2 activity.

## MIRNAS TARGETING *PTPN11*

*PTPN11* encodes the SHP2 tyrosine phosphatase which cooperates with GRB2 to transduce the signal from activated growth-factor-receptor to promote the RAS-MAPK pathway activation. Kikkawa *et al*. identified *PTPN11* mRNA as a *bona fide* target of miR-489 [43]. MiR-489 was one of the most down-regulated miRNAs in hypopharyngeal squamous cell carcinoma (HSCC). Down-regulation of miR-489 and subsequent activation of the RAS-MAPK pathway mediated by an enhanced SHP2 activity could represent a critical oncogenic event in HSCC.

## MIRNAS TARGETING *SOS*

After recruitment of GRB2 and SHP2 mediated by growth factor binding to cell-surface receptors, the RAS-MAPK pathway progresses with activation of effectors such as SOS1 (Son of Sevenless1). SOS1 is a guanine nucleotide-exchange factor (GNEF) that catalyzes guanine nucleotides shifting from RAS and subsequent binding to GTP to form the active GTP-bound RAS conformation. MiR-124 was found down-regulated in human GBM and SOS1 mRNA 3′-UTR was directly targeted by miR-124 in an *in vitro* model [44].

## MIRNAS TARGETING *KRAS*

In the year 2000, let-7 was the second miRNA, after lin-4, to be discovered [45]. This initial work demonstrated that let-7 was required during the development of *Caenorhabditis elegans*. Soon afterwards, the same team showed that let-7 was highly conserved across evolution from nematode to human, suggesting a major function of miRNAs as regulators of gene expression [46]. Later, a seminal work demonstrated that let-7 hybridized to the *RAS* ortholog transcript 3′-UTR and negatively regulated its expression [47]. In human, the let-7 family represents a ubiquitously expressed miRNAs family including 12 members (let-7-a1, a2, a3, b, c, d, e, f1, f2, g, i, and miR-98). Since the different members of the let-7 family have similar or even identical seed sequences, they likely have overlapping sets of target mRNAs. However, these different members may have different functions depending on the cellular context of their expression.

Many tumor types exhibited down-regulation of let-7 family members’ expression [48]. Interestingly, this finding was not restricted to cancer cells but undifferentiated normal human embryonic stem cells also harbored low expression of let-7. Moreover, two independent groups reported the *in vivo* tumor-suppressive role of let-7 [49, 50]. Lin28 family members are small proteins that bind to RNA and are specifically expressed in undifferentiated cell. Lin28 binds to let-7 miRNAs and blocks their processing by Dicer ribonuclease, resulting in a low level of mature let-7 in undifferentiated cell types (both normal embryonic stem cells and cancer cells) [51]. Interestingly, when these self-renewing cells were induced to differentiate, let-7 expression increased, indicating that the balance Lin28/let-7 was involved in the regulation of the cellular equilibrium between stemness and differentiation.

Polymorphisms in *KRAS* 3′-UTR have been described to alter let-7 binding, resulting in *KRAS* overexpression and subsequent aberrant activation of the RAS-MAPK pathway. The LCS6 (Let-7 Complementary Sites 6) SNP (rs61764370) has been associated with an increased risk of lung cancer among people with a moderate smoking history [52]. This LCS6 SNP was also described as a biomarker of cancer treatment response in oral cancer [53, 54] and colorectal cancer [55, 56]. Finally, LCS6 SNP predicted improved response to anti-EGFR monoclonal antibody monotherapy in patients with metastatic colorectal cancer [57]. In addition to let-7, many others miRNAs have been described to target the *KRAS* oncogene.

MiR-181a was found down-regulated in oral squamous cell carcinoma (OSCC) and was demonstrated to target the 3′-UTR of *KRAS* mRNA [58]. A genome-wide analysis has revealed *HRAS* mutations in ~12% of OSCC [59]. Hence, down-regulation of miR-181a could represent an epigenetic alternative to mediate RAS-MAPK activation in OSCC with no *HRAS* oncogenic mutation. Other members of the miR-181 family (with a similar seed sequence) have also been described to target *KRAS* mRNA. MiR-181c was shown to directly target *KRAS* and exhibited a weak expression in gastric carcinoma [60]. MiR-181c expression was up-regulated after 5-aza-2′-deoxycytidine hypomethylating treatment, indicating that miR-181c may be silenced through methylation. Additional data showing a miR-181d down-regulation in human gliomas, confirmed that miR-181d acted as a tumor suppressor by directly targeting *KRAS* mRNA [61].

MiR-134 was also shown to directly target the *KRAS* transcript. Identification of miR-134 down-regulation in GBM was correlated with the RAS-MAPK pathway activation [62]. MiR-134 was found to be down-regulated in renal cell carcinoma (RCC) samples, while overexpression of miR-134 suppressed proliferation by triggering G1/G0 cell cycle arrest. Forced expression of miR-134 could also inhibit migration and invasion by blocking epithelial-to-mesenchymal transition in RCC cell lines [63].

MiR-126 was found to be one of the most down-regulated miRNAs in PDAC and to directly target *KRAS* 3′-UTR [64]. In human pancreatic cancers, miR-96 down-regulation correlated with an elevation of *KRAS* expression and *KRAS* was shown to be a direct target of miR-96 [65]. MiR-217 was down-regulated in 75% of PDAC and directly targeted *KRAS* mRNA [66]. Interestingly, *KRAS* oncogenic mutations was the most frequent molecular abnormalities in this type of tumor and occurs in more than 90% of PDAC [2]. MiR-126, miR-96, or miR-217 down-regulation could represent an additional epigenetic reinforcement of RAS-MAPK pathway activation in pancreatic tumors.

MiR-143 expression was found to target *KRAS* mRNA and to be down-regulated in colorectal cancer [67, 68]. Interestingly, miR-143 and miR-145 clustered on chromosome 5q33.1 and both displayed decreased expression profiles in many cancer types. *TP53* loss-of-function led to a decreased level of both miR-143 and miR-145 through attenuating the maturation processing [69]. In addition, RAS activation led to down-regulation of the miR-143/145 cluster through RREB1 (RAS-responsive element-binding protein) activation that represses the miR-143/145 cluster promoter [70]. These results suggested a complex oncogenic network involving various partners such as p53 oncogene, miR-143/145 cluster, and the RAS-MAPK pathway.

*KRAS* was found to be a direct target of miR-200c in breast and lung cancer cell lines [71]. MiR-216b was down-regulated in nasopharyngeal carcinoma and bound *KRAS* mRNA 3′-UTR. MiR-216b decreased expression is directly related to aggressive nasopharyngeal carcinomas making this miRNA a potential prognosis biomarker [72]. In a model of transformed human bronchial epithelial cell line, Han *et al*. demonstrated that miR-622 down-regulation was correlated with an activation of the RAS-MAPK pathway due to a direct interaction with *KRAS* mRNA [73]. MiR-433 was significantly down-regulated in gastric cancer and directly bound *KRAS* mRNA to repress its expression [74]. Interestingly, treatment with 5-aza-2′-deoxycytidine hypomethylating agent could restore the expression of miR-433 in gastric cancer cells. This result indicates that, as for miR-181c, miR-433 down-regulation is mediated through methylation epigenetic silencing. Subramani et al. observed that miR-768 was reduced in patient brain metastases compared to primary tumor tissues and that *KRAS* was a direct target of miR-768. Brain microenvironment could mediate miR-768 down-regulation to activate RAS-MAPK signaling pathway and promote brain metastasis [75]. MiR-27a was down-regulated in esophageal squamous cell carcinoma and esophageal carcinoma cell lines. MiR-27a bound to *KRAS* mRNA and inhibited its translation, acting as a tumor suppressor through inhibition of the RAS-MAPK pathway [76].

MiR-30b and miR-30c were down-regulated in colorectal cancer [77] and breast cancer [78], respectively. *KRAS* was validated as a direct target of miR-30b and miR-30c.

In a model of chemically induced mouse skin tumorigenesis, the miR-193b/365a cluster expression was significantly reduced during tumor progression. Both miR-193b and miR-365a could acted as a tumor suppressor in the epidermis by directly targeting *KRAS* oncogene [79]. These results confirmed that clustered miRNAs (i.e. transcribed as a unique multicistronic RNA transcripts) could target the same messenger, for example here, acting as synergistic co-regulators of the RAS-MAPK pathway.

MiR-206 was found to be abrogated in human PDAC and in PDAC cell lines [80]. MiR-206 directly targeted *KRAS*, thereby acting as tumor suppressor in PDAC cells by blocking cell cycle progression, cell proliferation, migration, and invasion. Re-expression of miR-206 in PDAC cells was sufficient to inhibit tumor blood and lymphatic vessel formation, thus leading to a significant delay of tumor growth and progression.

Many miRNAs targeting the *KRAS* oncogene were found to be down-regulated in human cancers, acting as tumor suppressors. Conversely, some miRNAs were up-regulated following *KRAS* oncogenic mutations. MiR-21 production was up-regulated by oncogenic *KRAS* in thyroid carcinomas, non-small-cell lung cancers [81, 82], laryngeal squamous cell carcinoma [83], and PDAC [84]. This illustrated another mechanism of how the RAS-MAPK pathway interplays with miRNAs machinery. Indeed, miR-21 was found to be an oncomiR that targeted several tumor suppressor gene including *PTEN* and the p53 network [82]. Interestingly, it was also demonstrated that miR-21 targeted antagonists of the RAS-MAPK pathway (*SPRY1*, *SPRY2*, *BTG2*, and *PDCD4*), resulting in the activation of this pathway [85]. MiR-155 was induced by *KRAS* oncogenic signal mediated by reactive oxygen species (ROS) generation in pancreatic cancer [86].

## MIRNAS TARGETING *NRAS*

Cimino *et al*. identified *NRAS* as being directly down-regulated by miR-148b, using luciferase assays. MiR-148b down-regulation in aggressive breast tumors was found to be involved in tumor cells invasion and chemotherapy resistance, suggesting miR-148b as a biomarker [87].

MiR-124 down-regulation was identified in GBM stem cells and tumors [88]. MiR-124 is composed of miR-124-1, -2, and -3, which are transcribed from three different chromosome locus, but have the same seed sequence and most likely the same targets. MiR-124 was experimentally shown to target *NRAS* in GBM. Interestingly, miR-124 was also demonstrated to target SOS1, another effector of the RAS-MAPK pathway [44]. Somatic loss-of-function mutations in the tumor suppressor *NF1*, leading to RAS-MAPK pathway activation, have been described in ~25 % of GBM [89]. MiR-124 down-regulation could mediate an alternative oncogenic event to promote RAS-MAPK activation in *NF1* wild-type GBM.

MiR-143 was also demonstrated to directly target *NRAS* and may function as a tumor-suppressor in glioma [90]. In human clinical specimens, miR-143 was down-regulated while *NRAS* was up-regulated. Overexpression of miR-143 decreased glioma cell migration, invasion, tube formation and slowed tumor growth and angiogenesis in a manner associated with *NRAS* down-regulation *in vitro* and *in vivo*.

MiR-146a was shown to be highly up-regulated by oncogenic *BRAF* and *NRAS* mutants frequently observed in melanomas. MiR-146a subsequently promotes initiation and progression of melanomas by activating Notch signaling [91]. Interestingly, it has also been demonstrated that miR-146a targets *NRAS* mRNA in an endothelial cells model [92]. This result suggested a negative feedback loop to prevent an aberrant activation of the RAS-MAPK pathway in a physiological context that can be overwhelmed in an oncogenic background. This observation illustrates the pleiotropic role of miRNAs with numerous targets and the complexity to reliably predict the effect of a specific miRNA dysregulation.

## MIRNAS TARGETING *HRAS*

It was demonstrated that miR-205 targeted *HRAS* oncogene and was down-regulated in prostate carcinoma. In this context, it has been demonstrated that miR-205 cooperated with miR-130a and miR-203 to activate the RAS-MAPK signaling pathway [93].

In addition to *KRAS*, it has been demonstrated that miR-134 also directly targeted *DHHC9* mRNA. DHHC9 catalysed HRAS palmitoylation, a post-translational modification required for membrane localization and HRAS accurate activity [94]. MiR-134 down-regulation in cancer could consequently lead to RAS-MAPK pathway activation by mediating cooperative KRAS overexpression and HRAS activation.

## MIRNAS TARGETING *BRAF*

Once bound to GTP, RAS proteins function as regulators of a complex signaling network (the so-called MAPK cascade) including the activation of the RAF serine/threonine kinases: ARAF, BRAF, and RAF1 (alias CRAF). Although there have been few evidences of miRNA directly targeting *BRAF* mRNA, it has been demonstrated that *BRAF* oncogenic mutations were associated with miRNAs deregulation in many cancer types. In papillary thyroid carcinoma [95] and melanoma [96] *BRAF* oncogenic mutations were highly prevalent and led to deregulation of several miRNAs involved in tumor formation. MiR-146b expression level was significantly higher in papillary thyroid carcinoma with *BRAF* mutation and significantly associated with invasive behavior [97]. MiR-146a (another miR-146 family member) was highly up-regulated by oncogenic *BRAF* and *NRAS* mutations in melanomas [91]. MiR-193a, miR-338, and miR-565 were down-regulated in melanomas with *BRAF* mutations [98]. Altogether, oncogenic *BRAF* V600E mutation in melanoma cells was shown to control a network of >20 miRNAs with combinatorial functions to modulate the expression of key cancer regulatory genes [96]. MiR-31-5p was one the most up-regulated miRNA in colorectal cancers with *BRAF* p.V600E oncogenic mutation, compared with wild-type *BRAF* and play a role in cell invasion and proliferation in this tumor type [99].

A single miRNA can directly target several members of the RAS-MAPK pathway, acting in a synergistic effect. MiR-524-5p was shown to directly bind to the 3′-UTR of both *BRAF* and *ERK2* and to suppress the expression of these proteins in melanoma cells [100]. Because BRAF and ERK2 are main components of the RAS-MAPK pathway, the decreased expression of miR-524-5p in melanomas could mediate tumor proliferation and cell migration in melanomas.

## MIRNAS TARGETING *ARAF*

MiR-34a is regarded as a tumor suppressor regulating hundreds of transcripts including RAS-MAPK pathway members. MiR-34a was experimentally validated to repress *ARAF* expression [101]. MiR-34a expression was down-regulated in many tumor types by promoter methylation [102].

## MIRNAS TARGETING *RAF1* (ALIAS *CRAF*)

MiR-7 directly targeted *RAF1* mRNA and subsequently down-regulated its expression in lung, breast, and GBM cancer cell lines [103]. Interestingly, miR-7 also bound *EGFR* mRNA 3′-UTR that encodes an upstream members of the RAS-MAPK pathway [25, 103]. This is another example that miRNAs can target several members of a same pathway to mediate a synergistic oncogenic effect.

## MIRNAS TARGETING *MEK1*

MAP kinase kinases MEK1 and MEK2 (alias MAP2K1 and MAP2K2) act downstream of RAF serine/threonine kinases and phosphorylate MAP kinases ERK1 and ERK2. MiR-497 targeted and repressed *MEK1* expression in HeLa cells [104]. Interestingly, miR-497 was also associated with a decreased RAF1 and ERK1 protein levels. MiR-424 level was down-regulated in senile hemangioma, an abnormal proliferation of blood vessels compared to normal skin or other vascular anomalies [105]. *MEK1* mRNA was one of miR-424 targets and MEK1 increased protein expression related to miR-424 down-regulation could lead to abnormal cell proliferation in senile hemangioma. Prediction algorithms identified two miR-1826 binding sites in *MEK1* mRNA. *MEK1* expression was experimentally repressed by miR-1826. In addition, miR-1826 down-regulation was found in bladder cell lines and cancers and in von Hippel-Lindau (VHL)-inactivated renal cancer cells [106]. Interestingly, low miR-1826 expression was significantly associated with bad prognosis in this renal cancer subtype. MiR-181b directly bound to *MEK1* transcript 3′-UTR and contributed to chemoresistance in glioma [107]. In addition, *KRAS* was shown to be targeted by miR-181a in oral squamous cell carcinoma, by miR-181c in gastric carcinoma, and by miR-181d in glioma. These observations emphasized the role of miR-181 family as a major negative regulator of the RAS-MAPK pathway in cancer. Recently, *MAP2K4* targeted by miR-181a was shown to play a substantial role in pancreatic cancer invasion and progression [108].

## MIRNAS TARGETING *ERK1/ERK2*

MiR-483-5p was significantly down-regulated in gliomas and directly targeted *ERK1* (alias MAPK3) transcript [109]. MiR-124/214 cluster is down-regulated in squamous cell carcinoma. It has been demonstrated that miR-214 targeted *ERK1* transcript whereas *ERK2* (alias MAPK1) was regulated by miR-124 and miR-214 [110]. These results gave some interesting insight into combined mechanism by which clustered miRNAs synergistically regulates their targets.

## MIRNAS TARGETING *SPRED1* AND/OR *RASA1*

*SPRED1* encodes a member of the Sprouty family which inhibits the RAS-MAPK pathway. SPRED1 can associate with neurofibromin, the *NF1* gene product, to mediate its membrane localization and subsequent RAS inhibition [111]. *RASA1* (*RAS p21 protein activator GTPase activating protein 1*) encodes a GTPase-activating protein (GAP) that stimulates RAS GTPase activity, thus acting as a suppressor of the RAS-MAPK pathway. Hence, both SPRED1 and RASA1 function as negative regulators the RAS-MAPK signaling pathway. It has been demonstrated that miR-206 and miR-21 repressed the expression of both *RASA1* and *SPRED1* by targeting their mRNA 3′-UTRs in triple-negative breast cancer [112].

MiR-132 was highly expressed in many human cancer types but its overexpression would be restricted to the tumor vascular endothelium. MiR-132 promotes neovascularization by directly suppressing endothelial *RASA1* expression, leading to the RAS-MAPK pathway activation in endothelial cells [113]. Since induction of angiogenesis is a hallmark of cancer, initiation of neovascularization mediated by miRNAs deregulation emphasizes the importance of miRNAs in tumor formation by a mechanism different from merely enhancing proliferation. MiR-31 was one of the most significantly up-regulated miRNAs in colorectal cancer. MiR-31 directly targeted *RASA1* and hereafter activated the RAS-MAPK signaling pathway [114].

## MIRNAS TARGETING *NF1*

*NF1* encodes neurofibromin, a negative regulator of the RAS-MAPK pathway. MiR-10b was up-regulated in malignant peripheral nerve sheath tumors (MPNSTs) [115] and directly targeted the *NF1* mRNA [116]. Mavrakis et al. identified a subset of miRNAs involved in the development of T-cell acute lymphoblastic leukemia, that included miR-27a targeting the *NF1* mRNA [117]. MiR-370 was up-regulated in acute myeloid leukemia and functional analyses showed that miR-370 directly targeted *NF1* mRNA [118]. Interestingly, *NF1* gene deletions and mutations are frequent oncogenic events in acute myeloid leukemia [119] and MPNST. Hence, up-regulation of miRNAs repressing the *NF1* gene could represent an alternative oncogenic event to activate the RAS-MAPK pathway in these neoplasms.

MiR-9 is encoded by three distinct genes in human (i.e. miR-9-1, miR-9-2, and miR-9-3). MiR-9 was highly expressed in glioma and promoted the invasion of glioma cells by directly targeting *NF1* messenger [120]. MiR-193b was overexpressed in HNSCC, and directly targeted the *NF1* mRNA. Interestingly, activation of the RAS-MAPK pathway mediated by miR-193b up-regulation correlated with a lower disease-free survival and could serve as a novel prognostic biomarker in HNSCC [121].

## MIRNAS AS A RHEOSTAT OF THE ONCOGENIC RAS MAPK

Activation of the RAS-MAPK pathway is one of the most frequent oncogenic events in human cancer. Oncogenic RAS mutations occur in approximately 30% of tumors; however, mutations in upstream regulators and downstream effectors are also highly prevalent in many tumor types. Irrespective of the etiology, the oncogenic RAS signal is frequently potentiated as cancers progress through amplification of mutant *RAS* genes or suppression of negative feedback pathways. Epigenetic is likely to contribute to this emerging aspect of tumor evolution. Many miRNAs have been described to stimulate the RAS-MAPK pathway in many cancer types, acting as a rheostat of the oncogenic RAS signal that is increased as cancers progress. Specific mechanisms leading to miRNAs deregulation and their functional consequences in cancer are far from being fully elucidated. Since one miRNA can regulate thousands of genes and hundreds of miRNAs can repress the same transcript, effective prediction of miRNA targets remains particularly challenging. Reliability of computational tools is not completely valid. Integration of the different single miRNAs variations and targets of the RAS-MAPK pathway in human cancers is also challenging. Bioinformatic tools for data integration (miRNAome), interpretation and representation from individual miRNA expression variation are needed. Moreover, miRNAs expression variations in cancer should be cautiously interpreted, as correlation may be different from causality.

The cellular context should be taken into account and miRNAs deregulation consequences should be experimentally validated using *in vitro* or *in vivo* models. Here we provide a comprehensive map of miRNAs which have been experimentally proven to target members of the RAS-MAPK pathway. Dysregulation of miRNAs that target the RAS-MAPK pathway can represent an oncogenic event leading to sustained proliferation. MiRNAs can act either as oncogenes (OncomiRs) or tumor suppressors. OncomiRs function to suppress the expression of tumor suppressor genes [122]. Conversely, tumor suppressive miRNAs, such as let-7 and miR-34, have been found to repress the expression of oncogenes, such as *KRAS*.

From a clinical point of view, miRNAs could serve as diagnostic and prognostic biomarkers [123] and circulating miRNAs in plasma may be useful for early detection of cancer [124, 125]. MiRNA-based predictive biomarkers hold promise to inform about the probability of response rates in cancer. In colorectal cancer patients, LSC6 polymorphism in the let-7 binding site of the *KRAS* gene, has been proposed to predict the tumor responsiveness in EGFR-directed (cetuximab) treated patients [126].

Finally, the function of miRNAs can be efficiently and specifically inhibited by chemically modified antisense oligonucleotides, supporting their potential as targets for the development of novel therapies [127]. Mimetic miRNAs (mimic) with tumor suppressor properties could have a therapeutic impact. For example, *in vivo* treatment with miR-34a prevented tumor formation and progression in a pre-clinical transgenic therapeutically resistant *Kras^LSL-G12D/+^;Trp53^LSL-R172H/+^* mouse lung cancer model [128]. Another study suggested that the delivery of let-7 miRNA into tumors may have therapeutic benefit in patients with cancer and mimic are currently developed [49, 50]. MiRNA-targeting therapies are an area of intense interest to pharmaceutical companies, and many such compounds are in preclinical and clinical development (https://clinicaltrials.gov/ct2/show/NCT01829971?term=Mirna+Therapeutics&rank=1). A better understanding of the contribution of miRNAs to RAS-MAPK-driven cancers will help to guide future studies and will provide a scientific rationale for developing new therapies for still untreatable tumors.
